# Association between fasting glucose and all-cause mortality according to sex and age: a prospective cohort study

**DOI:** 10.1038/s41598-017-08498-6

**Published:** 2017-08-15

**Authors:** Sang-Wook Yi, Sangkyu Park, Yong-ho Lee, Hyang-Jeong Park, Beverley Balkau, Jee-Jeon Yi

**Affiliations:** 1 Department of Preventive Medicine and Public Health, Catholic Kwandong University College of Medicine, Gangneung, 25601 Republic of Korea; 2 Institute for Clinical and Translational Research, Catholic Kwandong University College of Medicine, Gangneung, 20601 Republic of Korea; 3 Department of Biochemistry, Catholic Kwandong University College of Medicine, Gangneung, 25601 Republic of Korea; 4 Division of Endocrinology and Metabolism, Department of Internal Medicine, Yonsei University College of Medicine, Seoul, 03722 Republic of Korea; 5 Department of Health Promotion, National Health Insurance Service, 26464 Wonju, Republic of Korea; 6 Center for Research in Epidemiology and Population Health (CESP), Team 5 (EpReC, Renal and cardiovascular Epidemiology), INSERM U-1018, 94807 Villejuif, France; 7 Institute for Occupational and Environmental Health, Catholic Kwandong University, Gangneung, 25601 Republic of Korea

## Abstract

The association of fasting glucose with the risk of death according to sex and age remains unclear, and insufficient information is available on sex- and age-specific glucose concentrations within ethnic groups. This study analyzed a sample of 12,455,361 Korean adults who participated in health examinations during 2001–2004, and were followed up until 2013. Men had 3.0 mg/dL (0.167 mmol/L) higher mean glucose concentrations than women (94.7 vs. 91.7 mg/dL), although women over 73 years had higher levels. For glucose levels of 100–199 mg/dL, each 18 mg/dL (1 mmol/L) increase in fasting glucose increased mortality by 13% (HR = 1.13, [95% CI 1.12 to 1.13], p < 0.001). In individuals with fasting glucose levels of 100–125 mg/dL, each 18 mg/dL increase in fasting glucose was associated with a 30% increase in the risk for mortality (1.30, [1.18 to 1.43]) in those aged 18–34 years, a 32% increase (1.32, [1.26 to 1.39]) in those aged 35–44 years, and a 10% increase (1.10, [1.02 to 1.19]) in those aged 75–99 years. The fasting glucose levels associated with the lowest mortality were 80–94 mg/dL regardless of sex and age. Prediabetes (100–125 mg/dL) was associated with higher mortality. The associations of hyperglycemia with mortality were stronger at younger ages.

## Introduction

Diabetes is a worldwide epidemic. Fasting glucose measurement remains the main test for the diagnosis of diabetes and prediabetes, and it is a key indicator of future diabetes and cardiovascular disease^1, 2^. However, the current cut-points, for example, 100 or 110 mg/dL for prediabetes, are somewhat arbitrarily chosen due to inconsistent results in prior studies^1–6^. Identifying proper cut-points for fasting glucose and overall mortality would help inform public and clinical actions to prevent premature death from health issues related to hyperglycemia and hypoglycemia.

Although fasting glucose levels are generally known to increase with advancing age^7–9^ and may differ by sex^8–10^, the sex- and age-specific associations of fasting glucose with all-cause mortality have rarely been examined. It is unclear whether variation according to age and sex is present in the association between fasting glucose and the risk of death and/or the range of fasting glucose associated with a minimal risk of death^7, 11, 12^. Additionally, age- and sex-specific fasting glucose concentrations may differ across ethnic and regional groups^8, 9, 13^, meaning that the association of fasting glucose levels with diseases and mortality may also vary by ethnicity^14^. Furthermore, detailed sex- and age-specific fasting glucose concentrations in the Korean population have not been reported.

Through a large prospective cohort study that included 12.8 million participants, we set out to elucidate whether the association between fasting glucose levels and all-cause mortality varies by sex and age, and to estimate sex- and age-specific levels of fasting glucose associated with minimal mortality. Precise estimates could help determine cut points for the management of hyperglycemia and hypoglycemia. Additionally, detailed estimates of mean (and median) concentrations of fasting glucose according to sex and age could help in the management of sex- and age- specific aspects of glucose metabolism and related metabolic disorders.

## Methods

### Study population and follow-up

The National Health Insurance Service (NHIS) provides compulsory health insurance that covers 97% of the Korean population. The Korean Metabolic Risk factor (KOMERIT) study was designed primarily to assess the risk of death associated with metabolic risk factors, and includes 12,845,017 NHIS beneficiaries aged 18–99 years who participated in routine health examinations during 2001–2004^15^. Individuals with missing information on serum glucose, blood pressure, total cholesterol, and body-mass index (BMI) were excluded (n = 26,136), as were 3,665 persons with extremely low weight (<30 kg), high BMI (≥50 kg/m^2^), or short stature (<1.30 m in persons below 55 years, <1.10 m for those aged 55 years or older), and another 210 missing date of the health examination. Persons with self-reported diabetes (n = 369,645) were also excluded. The final study population included 12,455,361 participants, followed up until the 31st December 2013 through the NHIS database of beneficiary status, with participants’ deaths being ascertained from the Resident Register of Korea^15^. All data are collected and maintained by the NHIS according to several Korean laws. For the research in accord with the conditions documented at Korean laws, health examination data can be provided to the researchers by the NHIS without specific informed consents from the participants. This study was approved by Institutional Review Board of Catholic Kwandong University, Republic of Korea. Anonymized data were provided to the authors by the NHIS, and they were only available through a specific computer within the NHIS headquarters.

### Data collection

Fasting serum glucose and total cholesterol were assayed using enzymatic methods. Blood pressure was measured in a seated position using a standard mercury sphygmomanometer. Weight and height were measured to the nearest kg and cm, respectively^15^. BMI was calculated by weight in kilograms divided by the square of height in meters (kg/m^2^). Smoking history, alcohol use, and known diabetes status were self-reported via questionnaire. Health examination and data collection followed a standard protocol, the Health Examination Practice Guide, officially registered by the Ministry of Health and Welfare (Health Examination Practice Guide [Korean]. External quality assessment in clinical chemistry, such as fasting glucose measurement, for hospitals was supervised by the Korean Association of Quality Assurance for Clinical Laboratory, and assessments of the quality of assays were high^16^.

### Data availability

The data that support the findings of this study are available from NHIS^17^, but restrictions apply to the availability of these data, which were used under license for the current study, and so are not publicly available.

### Statistical analysis

For analysis, fasting serum glucose concentrations were mainly categorized into 16 groups (<65, 65–69, 70–74, 75–79, 80–84, 85–89, 90–94 [Reference], 95–99, 100–104, 105–109, 110–117, 118–125, 126–139, 140–169, 170–199, ≥200 mg/dL). Eight (or seven) groups (<70, 70–79, 80–94 [Reference], 95–99, 100–109, 110–125, ≥126 [126–179, ≥180 mg/dL]), were used in additional analysis to show the difference in hazard ratios (HRs) for sex- and age-specific groups. The glucose category with the lowest mortality in sex and age-adjusted analysis was used as the referent. Fasting glucose level was also analyzed as a continuous variable and HRs per 18 mg/dL (1 mmol/L), rather than 10 mg/dL, increase in fasting glucose were calculated for between-study comparison^3^.

HRs for various fasting glucose categories with respect to the reference group were calculated using Cox proportional hazards models stratified by age (years) at baseline (18–24, 25–34, 35–44, 45–54, 55–64, 65–74, 75–84, 85–99) after adjustment for age at baseline (continuous variable; within each age group), sex (when applicable), smoking status (current smoker, former smoker, never-smoker, and missing information [n = 627,788]), alcohol use (frequency; monthly or less, 2 days/month-2days/week, 3–7 days/week, and missing information [n = 487,379]), physical activity (at least once a weak; yes, and no), body-mass index (BMI; continuous variable), systolic blood pressure (continuous variable), serum total cholesterol (continuous variable). Age at baseline was stratified for analyses into six groups (18–35, 35–44, 45–54, 55–64, 65–74, 75–99 years). The HRs for a restricted cubic spline transformation of fasting glucose with 5 knots (70, 85, 100, 120, 140 mg/dL) with mortality in participants having fasting glucose ≤300 mg/dL, were also plotted. Subgroup analyses with varying categories of fasting glucose served as sensitivity analyses.

Sex- and age-standardized death rates per 100,000 person-years (the simple mean of the applicable sex- and age-specific rates in 28 sex/age groups at ages 18–24 years, 25–29 years, and up to 85–99 years, increasing by 5 year age groups, based on age attained during follow-up) was calculated for each fasting glucose group^15^. All p-values were two-sided. All analyses used SAS version 9.4 (SAS Institute Inc., Cary, NC, USA).

## Results

During 134.9 million person-years of follow-up of 12,455,361 people (43.1% women), 416,557 men and 215,832 women died. At baseline the mean (standard deviation) age was 44.0(14.1) years and the mean fasting glucose level was 93.4(27.5) mg/dL (Table 1), 4.4% had fasting glucose levels 126 mg/dL or higher. Fasting glucose levels generally increased across six age groups (Tables S1–S3). Higher glucose levels were generally associated with higher BMI, systolic blood pressure, and total cholesterol values (Table S2).

**Table 1:** Characteristics of participants (n = 12,455,361).

Characteristics	Classification	Total	Men	Women
Age, years	Mean (±SD)	44.0 ± 14.1	42.9 ± 13.2	45.5 ± 15.1
Fasting serum glucose, mg/dL	Mean (±SD)	93.4 ± 27.5	94.7 ± 28.8	91.7 ± 25.6
BMI, kg/m^2^	Mean (±SD)	23.5 ± 3.2	23.8 ± 3.0	23.0 ± 3.3
Systolic blood pressure, mmHg	Mean (±SD)	123.9 ± 17.2	126.1 ± 16.1	121.0 ± 18.1
Total cholesterol, mg/dL	Mean (±SD)	194.0 ± 48.7	194.0 ± 47.1	193.9 ± 50.8
Sex	Men	7,090,089(56.9)	7,090,089(100.0)	0(0.0)
	Women	5,365,272(43.1)	0(0.0)	5,365,272(100.0)
Fasting serum glucose, mg/dL	<70	285,924(2.3)	157,981(2.2)	127,943(2.4)
	70–79	2,381,520(19.1)	1,299,853(18.3)	1,081,667(20.2)
	80–89	3,576,623(28.7)	1,910,731(26.9)	1,665,892(31.0)
	90–99	3,159,827(25.4)	1,787,246(25.2)	1,372,581(25.6)
	100–125	2,505,235(20.1)	1,567,046(22.1)	938,189(17.5)
	126–169	373,640(3.0)	248,713(3.5)	124,927(2.3)
	≥170	172,592(1.4)	118,519(1.7)	54,073(1.0)
Smoking status	Current smoker	3,565,515(28.6)	3,397,467(47.9)	168,048(3.1)
	Never smoker	7,200,954(57.8)	2,438,776(34.4)	4,762,178(88.8)
	Former smoker	1,061,104(8.5)	979,690(13.8)	81,414(1.5)
	Missing	627,788(5.0)	274,156(3.9)	353,632(6.6)
Alcohol use	Monthly or less	5,886,404(47.3)	2,110,523(29.8)	3,775,881(70.4)
	2/week–2/month	4,883,807(39.2)	3,627,965(51.2)	1,255,842(23.4)
	3–7 days/week	1,197,771(9.6)	1,096,946(15.5)	100,825(1.9)
	Missing	487,379(3.9)	254,655(3.6)	232,724(4.3)
Physical activity at least once a week	No	7,466,247(59.9)	3,694,570(52.1)	3,771,677(70.3)
	Yes	4,989,114(40.1)	3,395,519(47.9)	1,593,595(29.7)
Age, years	18–34	3,711,950(29.8)	2,279,667(32.2)	1,432,283(26.7)
	35–44	3,214,771(25.8)	2,017,090(28.4)	1,197,681(22.3)
	45–54	2,633,280(21.1)	1,388,966(19.6)	1,244,314(23.2)
	55–64	1,745,729(14.0)	876,433(12.4)	869,296(16.2)
	65–74	913,306(7.3)	424,218(6.0)	489,088(9.1)
	75–99	236,325(1.9)	103,715(1.5)	132,610(2.5)

Abbreviation: BMI, body-mass index; SD, standard deviation.

The difference for each characteristic was tested using ANOVA and the chi-square test, and the p-value for each variable, including total cholesterol, was <0.0001. To convert glucose from mg/dL to mmol/L, multiply by 0.0555.

To convert cholesterol from mg/dL to mmol/L, multiply by 0.0259.

### Fasting glucose concentration according to sex and age

**Figure 1:**
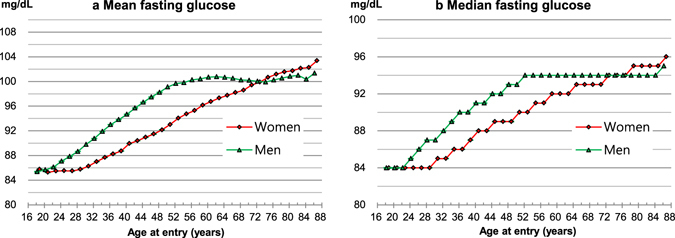
Mean and median concentration of fasting glucose. To convert glucose from mg/dL to mmol/L, multiply by 0.0555.

A unimodal distribution of fasting glucose was observed with a peak at 85–89 mg/dL (Figure S1). Men had 3.02 mg/dL (95% confidence interval [CI], 2.98 to 3.05) higher levels of fasting glucose than women. In men, the mean fasting glucose increased with age up until 62–63 years, while in women it did not substantially change up to 28–29 years and thereafter increased with age up to 86–99 years (Fig. 1, Table S3). Men and women had generally the same mean glucose levels at 18–23 years; thereafter, men had higher glucose levels up to around 72–73 years and women over 73 years had higher levels than men. The difference in glucose levels between sexes peaked at 48–51 years, when men had a 6.1 mg/dL higher mean glucose level compared with women.

### Associations between fasting glucose and mortality

**Figure 2:**
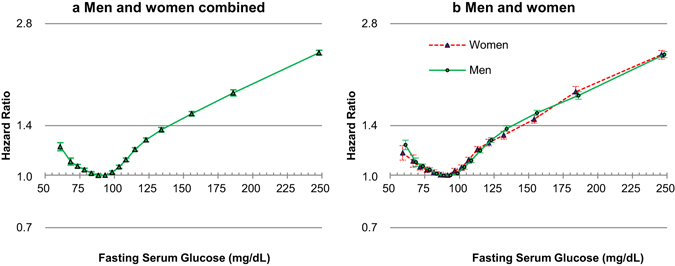
Hazard ratios^*^ associated with 16 categories of baseline fasting serum glucose (FSG) for mortality, according to sex. FSG categories (mg/dL: <65, 65–69, 70–74, 75–79, 80–84, 85–90, 90–94 [Reference], 95–99, 100–104, 105–109, 110–117, 118–125, 125–139, 140–169, 170–199, ≥200). The midpoint was used as a representative value for each FSG category, except for both ends (61 and 248), for which the median of all participants was used. *Hazard ratios and 95% confidence intervals were calculated using Cox proportional hazard models stratified by baseline age (years: 18–24, 25–34, 35–44, 45–54, 55–64, 65–74, 75–84, 85–99), after adjustment for age at baseline (continuous variable), sex (if applicable), smoking status, alcohol use, physical activity, body-mass index, systolic blood pressure, and total cholesterol levels. To convert glucose from mg/dL to mmol/L, multiply by 0.0555.

**Figure 3:**
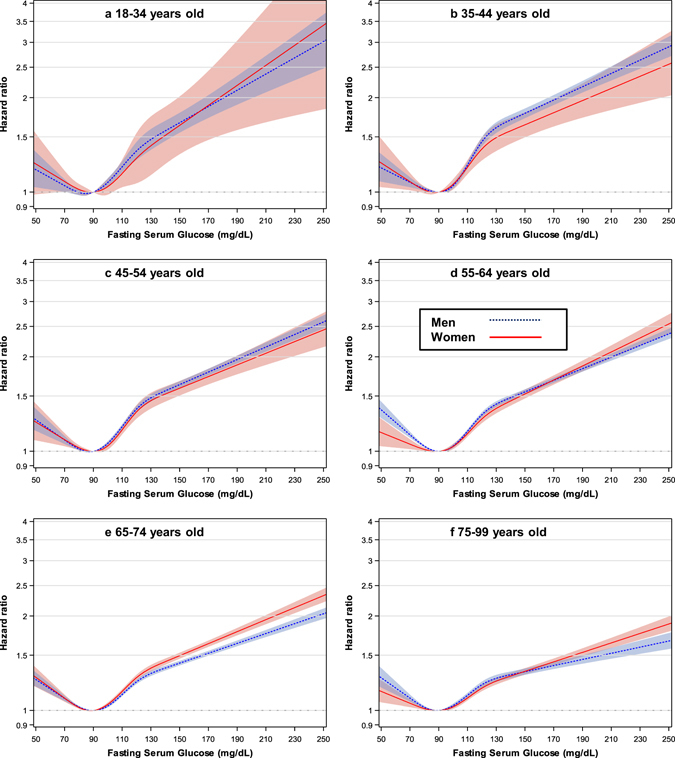
Hazard ratios* for mortality according to age by restricted cubic splines of fasting serum glucose with five knots (70, 85, 100, 120, and 140 mg/dL) and 90 mg/dL as a reference in men (n = 7,043,405) and women (n = 5,356,918) having fasting glucose ≤300 mg/dL. *Hazard ratios and 95% confidence intervals were calculated using the same method as in Fig. 2. To convert glucose from mg/dL to mmol/L, multiply by 0.0555.

J-curve-like associations between fasting glucose levels and mortality, with a nadir at around 80–94 mg/dL in both men and women (Fig. 2, Figure S5, Table S4) and in each sex/age group (Fig. 3. Table S5), were observed. HRs generally began to rise at fasting glucose levels around 95–100 mg/dL in each sex/age group, and began to rise from around 80 mg/dL and lower.

For glucose levels of 100–199 mg/dL assuming a log-linear relationship, the HRs per 18 mg/dL (1 mmol/L) increase in fasting glucose were 1.13 (95% CI, 1.12 to 1.13) in men and 1.12 (1.11 to 1.13) in women (Table 2). The associations were similar between sexes in all ages combined (P_interaction_ = 0.190), while women had stronger association than men in the age group of 65–74 years (HR per each 18 mg/dL increase = 1.13 in women vs. 1.11 in men, P_interaction_ = 0.012) in the sex- and age-specific analysis. For glucose levels below 100 mg/dL, the inverse associations between fasting glucose and mortality were stronger in men than in women (P_interaction_ = 0.014), especially in the age groups of 55–64 and 75–99 years.

**Table 2:** HRs^a^ per 18 mg/dL (1 mmol/L) FSG increase according to sex, age, and FSG range.

FSG range	Age group	Men			Women			p value for interaction between sexes
mg/dL	years	No. of death	p-value	HR (95% CI)	No. of death	p-value	HR (95% CI)	
100–199	All ages	149,904	<0.001	1.13 (1.12–1.13)	74,105	<0.001	1.12 (1.11–1.13)	0.190
	18–34	3,235	<0.001	1.15 (1.10–1.19)	491	0.060	1.12 (1.00–1.27)	0.737
	35–44	11,251	<0.001	1.17 (1.15–1.19)	2,031	<0.001	1.17 (1.12–1.23)	0.953
	45–54	22,746	<0.001	1.15 (1.14–1.17)	5,250	<0.001	1.13 (1.10–1.16)	0.255
	55–64	39,240	<0.001	1.14 (1.13–1.15)	13,030	<0.001	1.14 (1.13–1.16)	0.914
	65–74	48,086	<0.001	1.11 (1.10–1.12)	28,143	<0.001	1.13 (1.12–1.14)	0.012
	75–99	25,346	<0.001	1.08 (1.07–1.10)	25,160	<0.001	1.10 (1.08–1.11)	0.146
100–125	All ages	112,683	<0.001	1.20 (1.18–1.22)	56,842	<0.001	1.19 (1.16–1.22)	0.537
	18–34	2,823	<0.001	1.30(1.17–1.44)	454	0.118	1.25 (0.95–1.64)	0.769
	35–44	8,965	<0.001	1.31 (1.24–1.39)	1,770	<0.001	1.36 (1.20–1.56)	0.576
	45–54	17,138	<0.001	1.24 (1.20–1.29)	4,414	<0.001	1.32 (1.22–1.43)	0.195
	55–64	28,822	<0.001	1.21 (1.18–1.25)	10,248	<0.001	1.20 (1.14–1.26)	0.642
	65–74	35,940	<0.001	1.17 (1.14–1.20)	21,321	<0.001	1.19 (1.15–1.23)	0.369
	75–99	18,995	<0.001	1.12 (1.08–1.16)	18,635	<0.001	1.13 (1.09–1.18)	0.566
<100	All ages	254,446	<0.001	0.95 (0.94–0.96)	136,371	<0.001	0.96 (0.95–0.98)	0.014
	18–34	12,584	0.917	1.00 (0.96–1.03)	4,051	0.150	0.95 (0.89–1.02)	0.227
	35–44	23,904	<0.001	0.95 (0.92–0.97)	8,167	0.482	0.98 (0.94–1.03)	0.197
	45–54	37,576	0.001	0.97 (0.95–0.99)	14,725	0.004	0.95 (0.92–0.98)	0.451
	55–64	61,212	<0.001	0.93 (0.92–0.95)	26,023	0.080	0.98 (0.95–1.00)	0.004
	65–74	78,387	<0.001	0.95 (0.93–0.96)	46,632	<0.001	0.95 (0.94–0.97)	0.592
	75–99	40,783	<0.001	0.95 (0.93–0.96)	36,773	0.038	0.98 (0.96–1.00)	0.019

CI, confidence interval; FSG, fasting serum glucose; HR, hazard ratio.

^a^HRs were calculated by Cox models stratified by age (baseline age, years: 18–24, 25–34, 35–44, 45–54, 55–64, 65–74, 75–84, 85–99), after adjustement for age at baseline, sex (if applicable), smoking status, alcohol use, physical activity, body mass index, systolic blood pressure, and total cholesterol.

To convert glucose from mg/dL to mmol/L, multiply by 0.0555.

**Figure 4:**
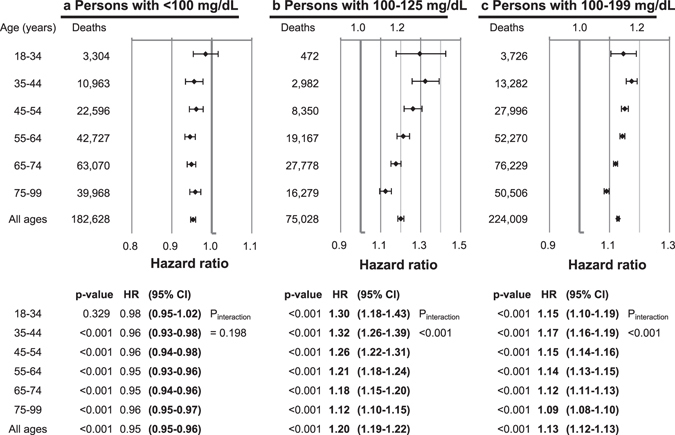
Hazard ratios^*^ per each 18 mg/dL (1 mmol/L) increase in fasting serum glucose (FSG), according to FSG range and age. *Hazard ratios and 95% confidence intervals were calculated using the same method as in Fig. 2. To convert glucose from mg/dL to mmol/L, multiply by 0.0555.

In the age-specific analysis for glucose levels of 100–199 mg/dL, the HRs per 18 mg/dL (1 mmol/L) increase in fasting glucose were 1.17 (95% CI, 1.16 to 1.19) in the age group of 35–44 years and 1.09 (95% CI, 1.08 to 1.09) in the age group of 75–99 years (Fig. 4). When the analysis was restricted to subjects whose levels were 100–125 mg/dL, the HRs per 18 mg/dL higher fasting glucose were 1.30 in those aged 18–34 years, 1.32 in those aged 35–44 years, and 1.12 in those aged 75–99 years. The HRs per 18 mg/dL higher fasting glucose were generally highest in the age group of 35–44 years and thereafter gradually decreased with age (P_interaction_ between age groups < 0.001 in both the 100–125 and 100–199 mg/dL ranges). The inverse associations in the range below 100 mg/dL were similar across age groups (P_interaction_ = 0.198).

## Discussion

Fasting glucose levels 80–94 mg/dL were associated with the lowest mortality regardless of sex and age, whereas glucose levels 100 mg/dL and above were clearly associated with higher mortality. Therefore, if the term ‘normoglycemia’ were to be used for glucose levels associated with the lowest mortality risk^2^, 100 mg/dL^18^ rather than 110 mg/dL^2^ might be a more appropriate upper limit of normoglycemia. Our age-stratified analysis showed that prediabetes ranges were relatively strongly associated with increased mortality, especially at younger ages (e.g., in men at 35–44 years, HR = 1.45, 95% CI, 1.37 to 1.54 in men with glucose levels 118–125 mg/dL compared to men with 90–94 mg/dL; Table S5). Overall, in the range of 100 mg/dL or above, HRs per increasing fasting glucose levels were generally stronger in younger adults than in older adults. In the lower glucose range, there was evidence that the risk of mortality starts increasing from around 80 mg/dL in line with previous research in Koreans^4^, but this may be discordant with a collaborative study in which levels of 70–100 mg/dL were not associated with higher risk^3^, however the population studied was much smaller than our population.

Fasting glucose levels generally increased with age and were higher in men compared with women, consistent with previous research^8, 9^. Our analysis further showed that glucose levels are similar between sexes at 18–21 years and begin to rise in men around the early 20s compared to the late 20s in women. Men had higher mean glucose levels than women from the early 20s to the early 70s; thereafter, women had higher levels than men. The difference in glucose levels between sexes peaked at 48–51 years. A number of factors including sex hormones, visceral adiposity, and muscle mass have been reported to regulate glucose metabolism^19–22^. Sex- and age-specific changes in sex hormones^23^, muscle mass^24^, and body adiposity^25^ generally concur with the observed sex- and age-specific changes in fasting glucose in our study. For example, small difference in mean BMI in women 18–29 years (21.1 kg/m^2^ at 18–19 years and 20.9 kg/m^2^ at 28–29 years) is concordant with no increase in mean fasting glucose. Furthermore, a well-known sharp decline in estrogen around the time of menopause corresponds with a sharp increase in fasting glucose in women around 50 years in the present study.

A recent systematic review showed that impaired fasting glucose as defined by the American Diabetes Association (IFG-ADA, fasting glucose of 100–125 mg/dL) was associated with higher mortality by 13% (relative risk [RR] = 1.13, 95% CI, 1.02 to 1.25) in all eligible studies, and by 9% (1.09, 0.97 to 1.22) in smoking-adjusted studies^26^. In this systematic review, IFG-ADA was associated with higher mortality in younger persons with an average age <55 years (RR = 1.28, 95% CI = 1.13 to 1.46), and in studies in which individuals with baseline cardiovascular disease were not excluded (1.19, 1.03 to 1.38), whereas such an association was not found in older persons (age ≥55 years) (1.04, 0.96 to 1.13) or in studies that excluded individuals with baseline cardiovascular disease (1.02, 0.86 to 1.19). In our study, the multivariate, smoking status-adjusted HRs associated with IFG-ADA were 1.12 (1.11 to 1.13) compared to normoglycemia (70–99 mg/dL) (Table S6). Although the associations of IFG-ADA were generally greater at younger ages (e.g., HR at 35–44 years = 1.17; 95% CI, 1.14 to 1.20), the oldest persons, aged 75–99 years at baseline, also had a higher mortality associated with IFG-ADA (1.08, 1.07 to 1.10) in the current study. Additionally, excluding persons with comorbid heart diseases or stroke (n = 137,708) only changed the association of IFG-ADA with mortality very slightly (1.12, 1.11 to 1.13; identical to 3 decimal places between with vs. without exclusion), with similar findings also observed for other glucose categories. Overall, our study provided a much more precise estimation of relative risk associated with IFG than the previous systematic review.

Considering the fairly strong relative risk associated with prediabetes (100–125 mg/dL), younger individuals with prediabetic glucose levels (particularly 110–125 mg/dL), should be carefully managed to prevent premature death. Individuals whose glucose is below 80 mg/dL (especially below 70 mg/dL) should be followed clinically.

Three large clinical trials in diabetic populations, failed to show a beneficial effect of intensive glycemic control on overall mortality and major vascular complications^27–30^, whereas the Diabetes Control and Complications Trial (DCCT)/the Epidemiology of Diabetes Interventions and Complications (EDIC) study, and the follow-up study in the United Kingdom Prospective Diabetes Study (UKPDS) demonstrated that better glycemic control reduced vascular complications and all-cause mortality^31, 32^. The participants in the DCCT/EDIC and UKPDS were younger and had less pre-existing cardiovascular complications than trials that showed no beneficial effect of intensive glycemic control on mortality. Our analysis showed that the estimated relative risk associated with increasing fasting glucose levels generally weakened with advancing age. These results suggest that glucose lowering interventions may be more beneficial for younger persons with fewer vascular complications^33^. In this regard, glycemic management in younger adults with prediabetic hyperglycemia (as well as those with diabetes) may be beneficial for preventing premature death.

The very large number of people and the complete follow-up for death are clear strengths of the study; however, its limitations are to be considered. Causal inference may be limited because of the observational nature of the study. Information on cause-specific mortality was not available due to privacy issues. Other measures of glucose tolerance such as postprandial glucose and HbA1c were also unavailable. Some potential confounders including socioeconomic status and dietary factors were not adjusted for in the main analysis. Including a socioeconomic status variable (decile of National Health Insurance premium that is determined by income and assets^34^; <4^th^ decile, 4^th^-7^th^ decile, ≥8^th^ decile) in the subpopulation analysis, however, showed no change in the findings. Nevertheless, unadjusted and residual confounding might affect the study results. Information on fasting glucose and other variables might vary in quality depending on the time and the hospitals. However, since the potential variability in data quality by hospitals would most likely be non-differential according to mortality, it would be unlikely to overestimate the associations. Nonetheless, this is a limitation of the study. Relative risk based on a single measurement of fasting glucose may underestimate the true association, due to a regression dilution effect^35^.

The fasting glucose range associated with the lowest mortality may be able to be generalized to other ethnic populations, since the range was generally the same across various sex and age groups that had varying cardiometabolic risk profiles. However, our study populations were generally leaner than other, more specifically European-origin, populations and had only a few smokers in women^15^. Further, accessibility to healthcare also affects the risk of complications, and deaths related to dysglycemia including diabetes^36^. Therefore, some results, such as the magnitude of relative risk associated with fasting glucose for all-cause mortality, may need to be assessed in other populations with varying distributions of risk factors, and varying access to healthcare utilization.

In conclusion, this large prospective cohort study showed a J-curve association, in which low and high fasting glucose concentrations were associated with higher mortality in the general population. The optimal fasting glucose levels for mortality (80–94 mg/dL) were generally the same regardless of sex and age, while the relative risk associated with increasing fasting glucose above those levels was greater at younger ages than at older ages. Prediabetic hyperglycemia was associated with excess mortality, especially in the range of 110–125 mg/dL.

## Electronic supplementary material

Supplementary information
