# Supplementary material for: Association between fasting glucose and all-cause mortality according to sex and age: a prospective cohort study
Source: Sci Rep. 2017 Aug 15;7:8194. doi: 10.1038/s41598-017-08498-6 (PMC5557842; doi:10.1038/s41598-017-08498-6)
Supplement: Supplementary file 1 — Supplementary information [file 41598_2017_8498_MOESM1_ESM.pdf]

Online-only Supplementary Data

Association between fasting glucose and all-cause mortality according to sex and age: a prospective cohort study.

Sang-Wook Yi, MD, PhD, Department of Preventive Medicine and Public Health, Catholic Kwandong University College of Medicine, Republic of Korea; **Sangkyu Park**, MD, PhD, Department of Biochemistry, Catholic Kwandong University College of Medicine, Republic of Korea; **Yong-Ho Lee**, MD, PhD, Division of Endocrinology and Metabolism, Department of Internal Medicine, Yonsei University College of Medicine, Republic of Korea; **Hyang-Jeong Park**, MSc, Department of Health Promotion, National Health Insurance Service, Republic of Korea; **Beverley Balkau**, PhD, Center for Research in Epidemiology and Population Health (CESP), Team 5 (EpReC, Renal and cardiovascular Epidemiology), INSERM U-1018, France; **Jee-Jeon Yi**, PhD, Institute for Occupational and Environmental Health, Catholic Kwandong University, Republic of Korea

Correspondence: Sang-Wook Yi, MD, PhD, Department of Preventive Medicine and Public Health, Catholic Kwandong University College of Medicine, Bumil-ro 579, Gangneung, Gangwon-do, 25601, Republic of Korea; Tel: +82-33-649-7468; Fax +82-33-641-1074. e-mail: flyhigh@cku.ac.kr

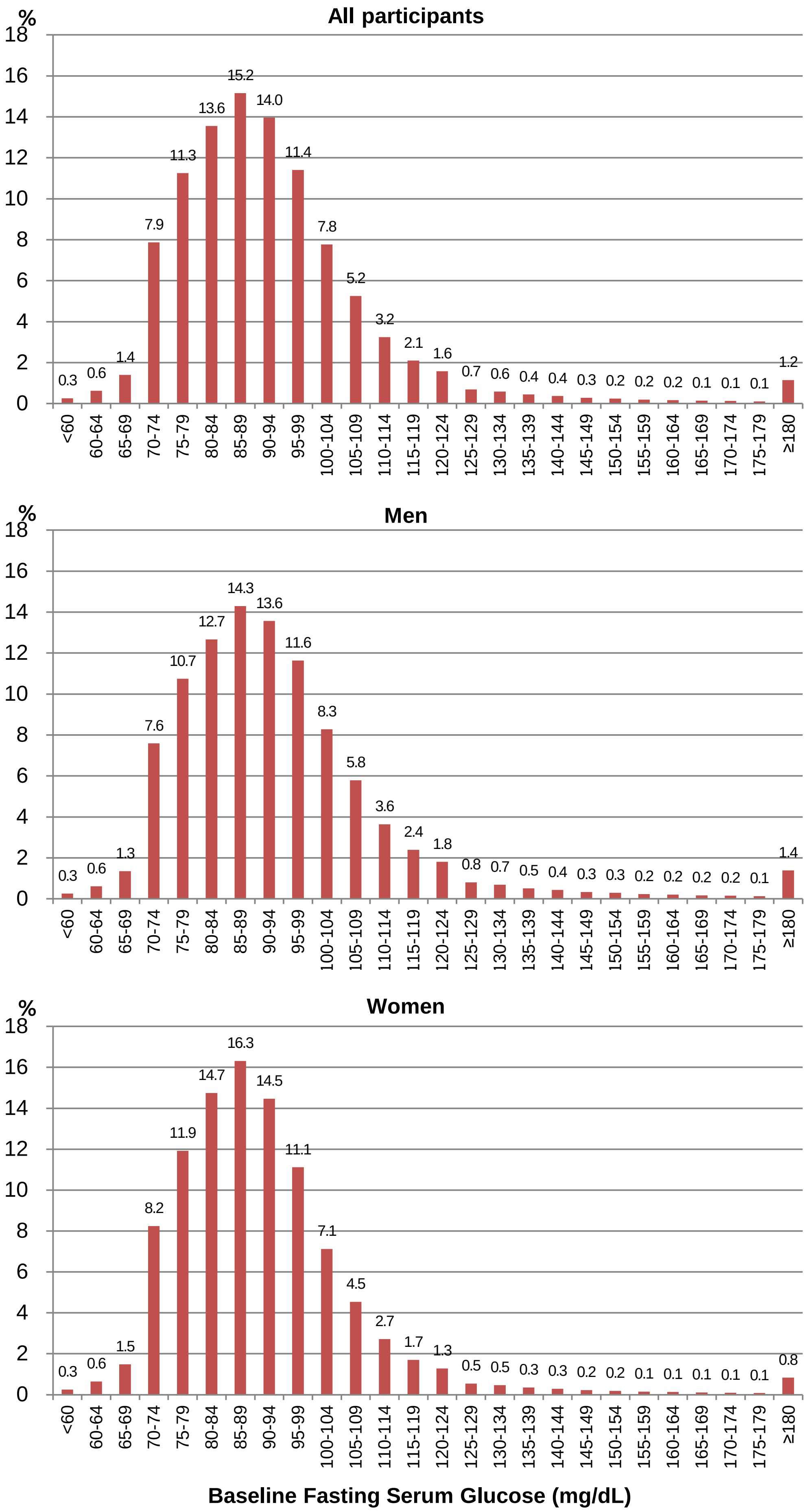

Figure S1. Distribution of fasting serum glucose concentration in Korean adults.
To convert glucose from mg/dL to mmol/L, multiply by 0.0555.

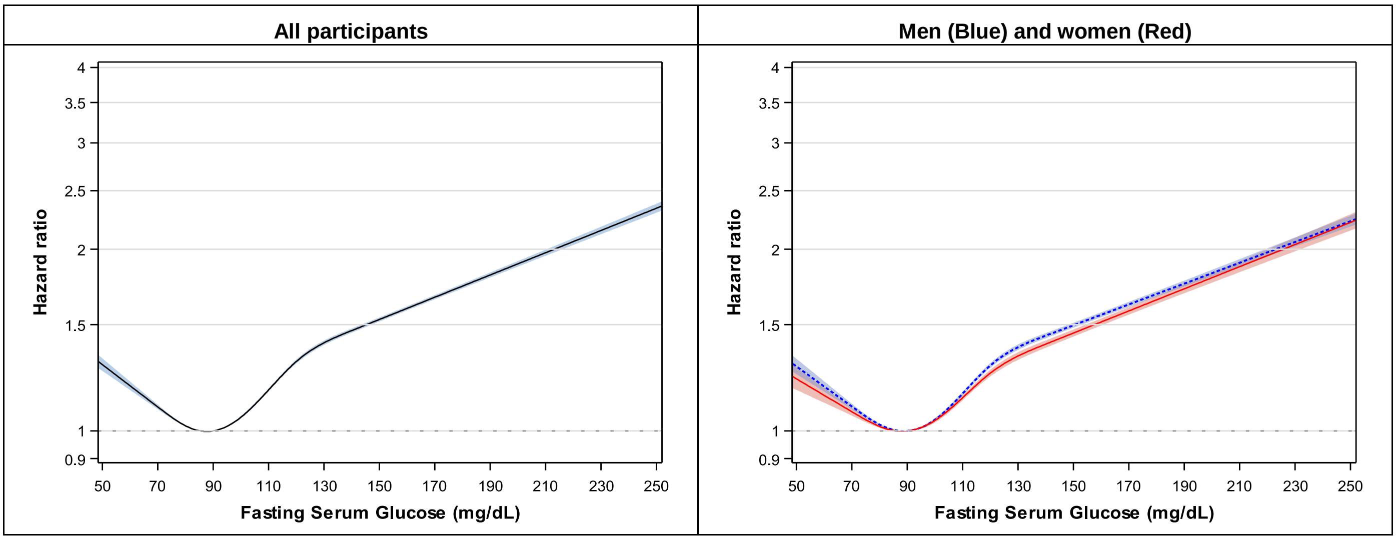

Figure S2. Hazard ratios[a] for all-cause mortality according to sex by restricted cubic splines of fasting serum glucose with five knots (70, 85, 100, 120, and 140 mg/dL) and 90 mg/dL as a reference in the participants having fasting glucose ≤ 300 mg/dL (n=12,430,323). [a]Hazard ratios and 95% confidence intervals were calculated using Cox hazards models after adjustment for age at baseline (continuous variable), sex (when applicable), smoking status, alcohol use, physical activity, body-mass index, systolic blood pressure, and total cholesterol levels. To convert glucose from mg/dL to mmol/L, multiply by 0.0555.

Table S1. Characteristics according to age groups in all participants.

Characteristics	Classification	18-99 years n = 12,455,361 n	(%)	18-34 years n = 3,711,950 n	(%)	35-44 years n = 3,214,771 n	(%)	45-54 years n = 2,633,280 n	(%)	55-64 years n = 1,745,729 n	(%)	65-74 years n = 913,306 n	(%)	75-99 years n = 236,325 n	(%)
Age, years	Mean (SD)	44.0	±14.1	28.0	±3.9	40.0	±2.8	49.3	±2.9	59.6	±2.9	68.9	±2.7	79.1	±3.5
Fasting serum glucose, mg/dL	Mean (SD)	93.4	±27.5	87.9	±21.0	92.7	±26.1	95.8	±29.4	98.5	±32.2	99.7	±33.7	101.4	±35.2
BMI, kg/m^2	Mean (SD)	23.5	±3.2	22.5	±3.3	23.7	±3.0	24.1	±2.9	24.2	±3.0	23.6	±3.2	22.6	±3.3
Systolic blood pressure, mmHg	Mean (SD)	123.9	±17.2	118.6	±13.6	121.2	±15.3	125.2	±17.4	130.7	±18.9	134.3	±19.8	137.3	±21.0
Total cholesterol, mg/dL	Mean (SD)	194.0	±48.7	181.7	±41.3	193.2	±45.3	201.6	±50.0	205.0	±54.2	202.7	±58.3	198.6	±56.4
Sex	Men	7,090,089	(56.9)	2,279,667	(61.4)	2,017,090	(62.7)	1,388,966	(52.7)	876,433	(50.2)	424,218	(46.4)	103,715	(43.9)
	Women	5,365,272	(43.1)	1,432,283	(38.6)	1,197,681	(37.3)	1,244,314	(47.3)	869,296	(49.8)	489,088	(53.6)	132,610	(56.1)
Smoking status	Current smoker	3,565,515	(28.6)	1,337,791	(36.0)	1,046,036	(32.5)	642,111	(24.4)	344,068	(19.7)	157,855	(17.3)	37,654	(15.9)
	Never smoker	7,200,954	(57.8)	1,886,639	(50.8)	1,707,160	(53.1)	1,623,778	(61.7)	1,176,528	(67.4)	638,972	(70.0)	167,877	(71.0)
	Former smoker	1,061,104	(8.5)	266,935	(7.2)	317,318	(9.9)	245,831	(9.3)	138,989	(8.0)	72,686	(8.0)	19,345	(8.2)
	Missing	627,788	(5.0)	220,585	(5.9)	144,257	(4.5)	121,560	(4.6)	86,144	(4.9)	43,793	(4.8)	11,449	(4.8)
Drinking status	Missing	487,379	(3.9)	176,647	(4.8)	108,904	(3.4)	86,368	(3.3)	68,339	(3.9)	37,006	(4.1)	10,115	(4.3)
	Monthly or less	5,886,404	(47.3)	1,284,852	(34.6)	1,361,571	(42.4)	1,372,481	(52.1)	1,069,585	(61.3)	623,335	(68.3)	174,580	(73.9)
	2/month-2/week	4,883,807	(39.2)	2,019,567	(54.4)	1,410,262	(43.9)	871,572	(33.1)	404,925	(23.2)	148,344	(16.2)	29,137	(12.3)
	3-7 days/week	1,197,771	(9.6)	230,884	(6.2)	334,034	(10.4)	302,859	(11.5)	202,880	(11.6)	104,621	(11.5)	22,493	(9.5)
Physical activity,	No	7,466,247	(59.9)	2,229,657	(60.1)	1,818,896	(56.6)	1,493,778	(56.7)	1,087,972	(62.3)	646,432	(70.8)	189,512	(80.2)
At least once a week	Yes	4,989,114	(40.1)	1,482,293	(39.9)	1,395,875	(43.4)	1,139,502	(43.3)	657,757	(37.7)	266,874	(29.2)	46,813	(19.8)
Fasting serum glucose, mg/dL	<65	110,963	(0.9)	48,521	(1.3)	25,018	(0.8)	17,273	(0.7)	11,366	(0.7)	6,973	(0.8)	1,812	(0.8)
	65-69	174,961	(1.4)	74,660	(2.0)	41,876	(1.3)	28,433	(1.1)	17,517	(1.0)	9,844	(1.1)	2,631	(1.1)
	70-74	980,137	(7.9)	401,221	(10.8)	242,981	(7.6)	168,615	(6.4)	99,996	(5.7)	53,618	(5.9)	13,706	(5.8)
	75-79	1,401,383	(11.3)	548,646	(14.8)	358,856	(11.2)	252,736	(9.6)	147,503	(8.4)	74,874	(8.2)	18,768	(7.9)
	80-84	1,688,724	(13.6)	603,698	(16.3)	441,780	(13.7)	326,532	(12.4)	195,371	(11.2)	97,651	(10.7)	23,692	(10.0)
	85-89	1,887,899	(15.2)	618,895	(16.7)	503,226	(15.7)	385,090	(14.6)	235,529	(13.5)	116,744	(12.8)	28,415	(12.0)

Characteristics	Classification	18-99 years n = 12,455,361 n	(%)	18-34 years n = 3,711,950 n	(%)	35-44 years n = 3,214,771 n	(%)	45-54 years n = 2,633,280 n	(%)	55-64 years n = 1,745,729 n	(%)	65-74 years n = 913,306 n	(%)	75-99 years n = 236,325 n	(%)
	90-94	1,738,336	(14.0)	506,822	(13.7)	470,033	(14.6)	377,961	(14.4)	237,037	(13.6)	117,856	(12.9)	28,627	(12.1)
	95-99	1,421,491	(11.4)	373,954	(10.1)	383,809	(11.9)	321,546	(12.2)	211,009	(12.1)	105,283	(11.5)	25,890	(11.0)
	100-104	968,673	(7.8)	216,062	(5.8)	257,400	(8.0)	230,668	(8.8)	160,206	(9.2)	83,392	(9.1)	20,945	(8.9)
	105-109	653,838	(5.2)	128,633	(3.5)	168,331	(5.2)	159,438	(6.1)	117,970	(6.8)	62,760	(6.9)	16,706	(7.1)
	110-117	564,899	(4.5)	95,178	(2.6)	137,307	(4.3)	141,044	(5.4)	111,418	(6.4)	62,557	(6.8)	17,395	(7.4)
	118-125	317,825	(2.6)	44,023	(1.2)	71,572	(2.2)	79,480	(3.0)	68,859	(3.9)	41,277	(4.5)	12,614	(5.3)
	126-139	195,850	(1.6)	22,677	(0.6)	40,895	(1.3)	49,825	(1.9)	45,645	(2.6)	28,092	(3.1)	8,716	(3.7)
	140-169	177,790	(1.4)	16,311	(0.4)	35,849	(1.1)	46,274	(1.8)	43,292	(2.5)	27,283	(3.0)	8,781	(3.7)
	170-199	70,377	(0.6)	5,051	(0.1)	14,220	(0.4)	19,384	(0.7)	17,741	(1.0)	10,669	(1.2)	3,312	(1.4)
	≥200	102,215	(0.8)	7,598	(0.2)	21,618	(0.7)	28,981	(1.1)	25,270	(1.4)	14,433	(1.6)	4,315	(1.8)

Header row 1: Age group

BMI, body-mass index; SD, standard deviation
To convert glucose from mg/dL to mmol/L, multiply by 0.0555.
To convert cholesterol from mg/dL to mmol/L, multiply by 0.0259.

Table S2. Mean of the metabolic mediators according to fasting serum glucose.

Age group,	FSG group,	Men and women combined				Men				Women			
			BMI, kg/m^2	SBP, mmHg	Total cholesterol, mg/dL		BMI, kg/m^2	SBP, mmHg	Total cholesterol, mg/dL		BMI, kg/m^2	SBP, mmHg	Total cholesterol, mg/dL
years	mg/dL	n	Mean (SD)	Mean (SD)	Mean (SD)	n	Mean (SD)	Mean (SD)	Mean (SD)	n	Mean (SD)	Mean (SD)	Mean (SD)
18-99	<65	110,963	22.7 (3.1)	120.7 (16.3)	184.3 (43.7)	62,538	23.1 (3.0)	123.5 (15.3)	184.4 (42.0)	48,425	22.2 (3.1)	117.0 (17.0)	184.3 (45.8)
all ages	65-69	174,961	22.7 (3.1)	120.4 (16.2)	185.1 (43.3)	95,443	23.2 (3.0)	123.3 (15.1)	185.5 (41.7)	79,518	22.2 (3.1)	116.9 (16.8)	184.6 (45.1)
	70-74	980,137	22.9 (3.1)	120.8 (16.2)	187.0 (44.2)	538,065	23.4 (3.0)	123.5 (15.1)	187.9 (43.1)	442,072	22.3 (3.2)	117.4 (16.8)	185.8 (45.5)
	75-79	1,401,383	23.0 (3.1)	121.0 (16.2)	188.1 (43.8)	761,788	23.5 (3.0)	123.7 (15.1)	189.1 (42.2)	639,595	22.4 (3.2)	117.7 (16.9)	186.9 (45.6)
	80-84	1,688,724	23.1 (3.1)	121.5 (16.4)	189.8 (43.8)	897,680	23.6 (2.9)	124.2 (15.3)	190.8 (42.7)	791,044	22.6 (3.2)	118.4 (17.0)	188.7 (44.9)
	85-89	1,887,899	23.3 (3.1)	122.2 (16.5)	191.7 (44.9)	1,013,051	23.7 (2.9)	124.7 (15.4)	192.1 (43.2)	874,848	22.8 (3.2)	119.4 (17.3)	191.1 (46.7)
	90-94	1,738,336	23.5 (3.1)	123.4 (16.7)	194.1 (45.4)	961,999	23.8 (2.9)	125.4 (15.6)	194.0 (43.0)	776,337	23.0 (3.2)	120.8 (17.7)	194.2 (48.2)
	95-99	1,421,491	23.7 (3.1)	124.6 (16.9)	196.3 (46.0)	825,247	24.0 (2.9)	126.3 (15.8)	195.6 (43.5)	596,244	23.3 (3.3)	122.3 (18.1)	197.2 (49.3)
	100-104	968,673	23.9 (3.1)	126.3 (17.3)	199.1 (55.7)	586,708	24.1 (3.0)	127.5 (16.2)	197.6 (53.2)	381,965	23.6 (3.3)	124.3 (18.6)	201.5 (59.3)
	105-109	653,838	24.1 (3.1)	127.7 (17.5)	201.1 (56.4)	410,578	24.2 (3.0)	128.7 (16.6)	199.2 (54.1)	243,260	23.9 (3.4)	126.1 (18.9)	204.3 (59.9)
	110-117	564,899	24.3 (3.2)	129.4 (17.8)	202.9 (57.7)	362,861	24.3 (3.0)	130.2 (16.9)	200.6 (54.7)	202,038	24.2 (3.4)	127.9 (19.3)	207.1 (62.6)
	118-125	317,825	24.4 (3.2)	130.9 (18.2)	203.9 (57.6)	206,899	24.4 (3.1)	131.5 (17.4)	201.2 (55.9)	110,926	24.5 (3.5)	129.7 (19.6)	209.1 (60.4)
	125-139	195,850	24.6 (3.3)	132.4 (18.7)	205.2 (56.3)	129,551	24.6 (3.1)	132.8 (17.9)	202.0 (54.3)	66,299	24.7 (3.5)	131.6 (20.1)	211.5 (59.6)
	140-169	177,790	24.7 (3.3)	133.3 (18.9)	206.5 (58.6)	119,162	24.6 (3.2)	133.6 (18.2)	203.1 (57.4)	58,628	24.8 (3.5)	132.6 (20.2)	213.4 (60.4)
	170-199	70,377	24.8 (3.3)	133.8 (19.0)	208.6 (60.5)	48,392	24.7 (3.2)	134.1 (18.5)	205.3 (59.0)	21,985	25.0 (3.6)	133.0 (20.2)	215.9 (63.2)
	≥200	102,215	24.6 (3.3)	133.6 (19.4)	218.0 (88.1)	70,127	24.5 (3.2)	133.9 (18.8)	214.9 (84.0)	32,088	24.7 (3.5)	132.9 (20.6)	225.0 (96.0)

BMI, body-mass index; FSG, fasting serum glucose; SBP, systolic blood pressure; SD, standard deviation

To convert glucose from mg/dL to mmol/L, multiply by 0.0555.

Table S3. Mean and median of the fasting serum glucose (mg/dL) (Including numerical version of Figure 1).

Age,	Men and women				Men				Women				Sex difference[a]	
years	n	Median	Mean	(SD)	n	Median	Mean	(SD)	n	Median	Mean	(SD)	Median	Mean
18-99	12,455,361	89	93.4	(27.5)	7,090,089	90	94.7	(28.8)	5,365,272	88	91.7	(25.6)	2	3.0
18-19	50,079	84	85.7	(19.2)	10,434	84	85.4	(15.1)	39,645	84	85.8	(20.2)	0	-0.4
20-21	167,615	84	85.4	(18.5)	55,725	84	85.7	(19.7)	111,890	84	85.3	(18.0)	0	0.4
22-23	326,869	84	85.7	(18.3)	111,568	84	86.1	(19.8)	215,301	84	85.5	(17.4)	0	0.6
24-25	495,402	85	86.2	(19.3)	206,009	85	87.1	(19.6)	289,393	84	85.5	(19.0)	1	1.6
26-27	564,398	85	86.8	(19.5)	321,754	86	87.8	(19.8)	242,644	84	85.5	(18.9)	2	2.3
28-29	617,749	86	87.8	(20.4)	430,135	87	88.7	(20.7)	187,614	84	85.8	(19.7)	3	2.9
30-31	607,008	87	88.9	(21.9)	456,018	87	89.8	(22.6)	150,990	85	86.3	(19.1)	2	3.5
32-33	583,368	88	89.9	(23.3)	453,377	88	90.7	(23.9)	129,991	85	87.0	(21.2)	3	3.7
34-35	540,608	88	91.0	(24.0)	421,081	89	91.9	(24.9)	119,527	86	87.7	(20.4)	3	4.2
36-37	504,017	89	91.9	(25.4)	390,691	90	93.0	(26.3)	113,326	86	88.3	(21.6)	4	4.7
38-39	484,268	89	92.6	(26.2)	370,178	90	93.8	(27.3)	114,090	87	88.7	(22.0)	3	5.1
40-41	761,812	89	92.6	(25.8)	423,900	91	94.7	(27.8)	337,912	88	89.9	(22.8)	3	4.7
42-43	738,803	90	93.4	(26.5)	412,301	91	95.7	(28.9)	326,502	88	90.4	(22.6)	3	5.3
44-45	681,315	90	94.0	(27.7)	370,434	92	96.6	(30.5)	310,881	89	91.0	(23.4)	3	5.7
46-47	624,363	91	94.7	(28.2)	333,754	92	97.5	(31.0)	290,609	89	91.5	(24.3)	3	6.0
48-49	557,533	91	95.3	(29.0)	288,397	93	98.2	(31.8)	269,136	89	92.2	(25.2)	4	6.1
50-51	488,314	91	96.1	(29.9)	250,952	93	99.1	(33.1)	237,362	90	93.0	(25.7)	3	6.1
52-53	436,421	92	96.9	(30.4)	225,353	94	99.7	(33.2)	211,068	90	94.0	(26.7)	4	5.6
54-55	430,571	92	97.4	(30.8)	223,337	94	99.8	(33.8)	207,234	91	94.7	(26.9)	3	5.1
56-57	370,509	93	97.8	(31.3)	186,665	94	100.3	(34.4)	183,844	91	95.3	(27.5)	3	5.0
58-59	327,034	93	98.3	(32.1)	163,686	94	100.4	(34.4)	163,348	92	96.2	(29.5)	2	4.3
60-61	359,246	93	98.7	(32.3)	177,341	94	100.7	(34.3)	181,905	92	96.7	(30.2)	2	3.9
62-63	335,113	93	99.0	(32.5)	161,496	94	100.8	(34.4)	173,617	92	97.3	(30.6)	2	3.5
64-65	298,159	93	99.2	(33.3)	143,642	94	100.7	(34.6)	154,517	93	97.7	(31.9)	1	2.9
66-67	257,825	93	99.3	(33.5)	121,774	94	100.5	(34.8)	136,051	93	98.2	(32.2)	1	2.3
68-69	219,300	93	99.3	(33.2)	102,337	94	100.2	(33.9)	116,963	93	98.6	(32.5)	1	1.7
70-71	170,418	94	99.8	(34.1)	77,587	94	100.2	(34.5)	92,831	93	99.4	(33.8)	1	0.8
72-73	130,783	94	99.9	(33.9)	57,070	94	100.0	(34.3)	73,713	94	99.9	(33.5)	0	0.1
74-75	103,685	94	100.3	(33.8)	44,964	94	99.9	(33.8)	58,721	94	100.7	(33.8)	0	-0.8
76-77	75,978	94	100.8	(35.4)	33,083	94	100.2	(35.1)	42,895	94	101.2	(35.7)	0	-0.9
78-79	53,483	94	101.1	(34.3)	23,982	94	100.5	(33.6)	29,501	95	101.6	(34.8)	-1	-1.0
80-81	39,643	94	101.3	(35.9)	17,658	94	100.8	(37.1)	21,985	95	101.7	(34.9)	-1	-0.9
82-83	24,476	95	101.6	(34.7)	10,905	94	101.0	(34.4)	13,571	95	102.1	(34.8)	-1	-1.1
84-85	13,780	95	101.4	(31.5)	6,165	94	100.4	(31.2)	7,615	95	102.3	(31.7)	-1	-1.9
86-99	15,416	96	102.5	(34.9)	6,336	95	101.3	(33.8)	9,080	96	103.4	(35.7)	-1	-2.0

SD, standard deviation.
[a] Concentration in men minus concentration in women.
To convert glucose from mg/dL to mmol/L, multiply by 0.0555.

Table S4. HRs[a] for death associated with fasting glucose, after adjustment for potential risk factors (Including numerical version of Figure 2).

sex	FSG group, mg/dL	Person year	No. of death	ASR[e]	Sex, and age adjusted[b] p-value	Sex, and age adjusted[b] HR (95% CI)	Additionally behavioral factors adjusted[c] p-value	Additionally behavioral factors adjusted[c] HR (95% CI)	Fully adjusted[d] p-value	Fully adjusted[d] HR (95% CI)
Men and Women	<65	1,192,954	5,635	2,064	<0.001	1.31 (1.27-1.34)	<0.001	1.26 (1.22-1.29)	<0.001	1.21 (1.18-1.25)
	65-69	1,878,971	7,503	1,871	<0.001	1.16 (1.14-1.19)	<0.001	1.13 (1.10-1.16)	<0.001	1.10 (1.07-1.12)
	70-74	10,458,930	39,102	1,828	<0.001	1.11 (1.10-1.13)	<0.001	1.09 (1.07-1.10)	<0.001	1.06 (1.05-1.08)
	75-79	14,945,216	53,540	1,774	<0.001	1.07 (1.06-1.08)	<0.001	1.06 (1.04-1.07)	<0.001	1.04 (1.03-1.05)
	80-84	17,939,000	65,942	1,697	<0.001	1.03 (1.02-1.05)	<0.001	1.02 (1.01-1.04)	0.018	1.01 (1.00-1.02)
	85-89	19,978,332	76,216	1,672	0.069	1.01 (1.00-1.02)	0.294	1.01 (1.00-1.02)	0.782	1.00 (0.99-1.01)
	90-94	18,345,353	75,204	1,657		1.00 (Reference)		1.00 (Reference)		1.00 (Reference)
	95-99	14,962,321	67,675	1,659	0.007	1.01 (1.00-1.02)	0.008	1.01 (1.00-1.02)	<0.001	1.02 (1.01-1.03)
	100-104	10,155,341	53,854	1,717	<0.001	1.05 (1.04-1.06)	<0.001	1.05 (1.04-1.06)	<0.001	1.06 (1.05-1.07)
	105-109	6,827,745	42,071	1,767	<0.001	1.10 (1.09-1.11)	<0.001	1.10 (1.08-1.11)	<0.001	1.11 (1.10-1.12)
	110-117	5,866,662	43,507	1,873	<0.001	1.18 (1.17-1.20)	<0.001	1.17 (1.16-1.19)	<0.001	1.19 (1.18-1.21)
	118-125	3,276,325	30,093	1,964	<0.001	1.27 (1.25-1.28)	<0.001	1.25 (1.23-1.27)	<0.001	1.27 (1.25-1.29)
	126-139	2,001,263	21,310	2,081	<0.001	1.36 (1.34-1.38)	<0.001	1.34 (1.32-1.36)	<0.001	1.36 (1.34-1.38)
	140-169	1,802,570	22,938	2,272	<0.001	1.53 (1.51-1.55)	<0.001	1.49 (1.47-1.52)	<0.001	1.52 (1.50-1.54)
	170-200	708,388	10,236	2,498	<0.001	1.76 (1.72-1.79)	<0.001	1.71 (1.68-1.75)	<0.001	1.75 (1.71-1.79)
	≥200	1,016,125	17,563	2,978	<0.001	2.31 (2.27-2.34)	<0.001	2.25 (2.21-2.29)	<0.001	2.30 (2.26-2.34)
Men	<65	671,180	3,920	2,771	<0.001	1.35 (1.31-1.40)	<0.001	1.29 (1.25-1.33)	<0.001	1.23 (1.19-1.27)
	65-69	1,025,428	5,029	2,376	<0.001	1.18 (1.15-1.21)	<0.001	1.13 (1.10-1.17)	<0.001	1.09 (1.06-1.12)
	70-74	5,753,320	26,184	2,381	<0.001	1.13 (1.11-1.15)	<0.001	1.09 (1.08-1.11)	<0.001	1.06 (1.05-1.08)
	75-79	8,147,381	35,271	2,307	<0.001	1.08 (1.07-1.10)	<0.001	1.06 (1.04-1.07)	<0.001	1.04 (1.02-1.05)
	80-84	9,566,606	42,537	2,184	<0.001	1.04 (1.03-1.05)	<0.001	1.03 (1.01-1.04)	0.121	1.01 (1.00-1.02)
	85-89	10,756,986	48,987	2,140	0.046	1.01 (1.00-1.03)	0.317	1.01 (0.99-1.02)	0.655	1.00 (0.98-1.01)
	90-94	10,188,727	48,506	2,117		1.00 (Reference)		1.00 (Reference)		1.00 (Reference)
	95-99	8,714,867	44,012	2,101	0.430	1.01 (0.99-1.02)	0.324	1.01 (0.99-1.02)	0.063	1.01 (1.00-1.03)
	100-104	6,165,166	35,683	2,194	<0.001	1.04 (1.03-1.06)	<0.001	1.05 (1.03-1.06)	<0.001	1.06 (1.04-1.07)
	105-109	4,295,599	27,980	2,253	<0.001	1.09 (1.07-1.11)	<0.001	1.09 (1.07-1.10)	<0.001	1.11 (1.09-1.12)
	110-117	3,774,615	28,958	2,368	<0.001	1.17 (1.15-1.18)	<0.001	1.16 (1.15-1.18)	<0.001	1.18 (1.16-1.20)
	118-125	2,136,369	20,062	2,510	<0.001	1.26 (1.24-1.28)	<0.001	1.25 (1.23-1.27)	<0.001	1.27 (1.25-1.29)
	126-139	1,326,024	14,392	2,654	<0.001	1.36 (1.33-1.39)	<0.001	1.34 (1.32-1.37)	<0.001	1.37 (1.35-1.40)
	140-169	1,209,792	15,741	2,905	<0.001	1.54 (1.51-1.57)	<0.001	1.50 (1.47-1.53)	<0.001	1.53 (1.50-1.55)
	170-200	487,727	7,088	3,115	<0.001	1.73 (1.69-1.77)	<0.001	1.69 (1.64-1.73)	<0.001	1.72 (1.68-1.76)
	≥200	697,032	12,207	3,702	<0.001	2.28 (2.24-2.33)	<0.001	2.22 (2.17-2.26)	<0.001	2.27 (2.22-2.31)

sex	FSG group, mg/dL	Person year	No. of death	ASR[e]	Sex, and age adjusted[b]		Additionally behavioral factors adjusted[c]		Fully adjusted[d]	
					p-value	HR (95% CI)	p-value	HR (95% CI)	p-value	HR (95% CI)
Women	<65	521,774	1,715	1,358	<0.001	1.21 (1.15-1.27)	<0.001	1.18 (1.13-1.24)	<0.001	1.16 (1.11-1.22)
	65-69	853,543	2,474	1,367	<0.001	1.14 (1.09-1.18)	<0.001	1.12 (1.07-1.17)	<0.001	1.10 (1.06-1.15)
	70-74	4,705,611	12,918	1,274	<0.001	1.08 (1.06-1.10)	<0.001	1.07 (1.05-1.09)	<0.001	1.06 (1.04-1.08)
	75-79	6,797,835	18,269	1,241	<0.001	1.06 (1.04-1.08)	<0.001	1.05 (1.03-1.07)	<0.001	1.04 (1.02-1.06)
	80-84	8,372,394	23,405	1,210	0.002	1.03 (1.01-1.05)	0.008	1.02 (1.01-1.04)	0.045	1.02 (1.00-1.04)
	85-89	9,221,345	27,229	1,203	0.478	1.01 (0.99-1.02)	0.523	1.01 (0.99-1.02)	0.717	1.00 (0.99-1.02)
	90-94	8,156,627	26,698	1,196		1.00 (Reference)		1.00 (Reference)		1.00 (Reference)
	95-99	6,247,454	23,663	1,218	0.002	1.03 (1.01-1.05)	0.004	1.03 (1.01-1.04)	0.001	1.03 (1.01-1.05)
	100-104	3,990,176	18,171	1,240	<0.001	1.05 (1.03-1.07)	<0.001	1.05 (1.03-1.07)	<0.001	1.05 (1.03-1.07)
	105-109	2,532,146	14,091	1,281	<0.001	1.10 (1.08-1.13)	<0.001	1.10 (1.08-1.12)	<0.001	1.11 (1.08-1.13)
	110-117	2,092,046	14,549	1,378	<0.001	1.19 (1.16-1.21)	<0.001	1.18 (1.16-1.20)	<0.001	1.19 (1.17-1.22)
	118-125	1,139,956	10,031	1,419	<0.001	1.24 (1.21-1.27)	<0.001	1.23 (1.20-1.26)	<0.001	1.25 (1.22-1.28)
	126-139	675,239	6,918	1,508	<0.001	1.31 (1.27-1.34)	<0.001	1.30 (1.26-1.33)	<0.001	1.31 (1.28-1.35)
	140-169	592,779	7,197	1,638	<0.001	1.46 (1.42-1.50)	<0.001	1.45 (1.41-1.48)	<0.001	1.46 (1.42-1.50)
	170-200	220,660	3,148	1,880	<0.001	1.75 (1.68-1.81)	<0.001	1.73 (1.67-1.80)	<0.001	1.76 (1.70-1.83)
	≥200	319,093	5,356	2,253	<0.001	2.25 (2.19-2.32)	<0.001	2.23 (2.16-2.29)	<0.001	2.27 (2.20-2.33)

ASR, age standardized death rate per 100,000 person-years; CI, confidence interval; FSG, fasting serum glucose; HR, hazard ratio

[a] HRs were calculated by Cox models stratified by age (baseline age, years: 18-24, 25-34, 35-44, 45-54, 55-64, 65-74, 75-84, 85-99), after adjustment for risk factors.

[b] Adjustement for age at baseline, and sex (if applicable)

[c] Adjustement for age at baseline, sex (if applicable), smoking status, alcohol use, and physical activity.

[d] Adjustement for age at baseline, sex (if applicable), smoking status, alcohol use, physical activity, body mass index, systolic blood pressure, and total cholesterol.

[e] Uniformly age-standardized death rate per 100,000 person-years (namely, the simple mean of calculable age-specific rates in the 14 age-specific rates at ages 18-24 years, and 25-29 years to 85 years or older by 5 years based on attained age during follow-up) was calculated for each FSG category. Several age-specific rates were calculated at each age group, and age-specific rate in the oldest ages at each age group can be very high due to small number of follow-up years. Therefore, death rate at a FSG category can be very high compared to other categories.

To convert glucose from mg/dL to mmol/L, multiply by 0.0555.

Table S5. HRs[a] for death associated with fasting glucose in men and women, according to age.

Age group, years	FSG group, mg/dL	Men (n=7,090,089)					Women (n=5,365,272)				
		Person year	No. of death	ASR[b]	p-value	HR (95% CI)	Person year	No. of death	ASR[b]	p-value	HR (95% CI)
18-34	<65	306,809	222	70	0.093	1.13 (0.98-1.29)	226,074	68	87	0.379	1.12 (0.87-1.44)
	65-69	459,120	284	65	0.641	0.97 (0.86-1.10)	357,353	105	30	0.440	1.09 (0.88-1.34)
	70-74	2,510,762	1,568	72	0.689	0.99 (0.92-1.05)	1,846,955	568	35	0.034	1.13 (1.01-1.27)
	75-79	3,430,299	2,082	77	0.159	0.96 (0.90-1.02)	2,522,360	753	44	0.096	1.10 (0.98-1.22)
	80-84	3,741,429	2,200	78	0.010	0.92 (0.87-0.98)	2,783,251	769	41	0.836	1.01 (0.91-1.13)
	85-89	3,932,918	2,403	71	0.149	0.96 (0.90-1.02)	2,727,931	808	38	0.163	1.08 (0.97-1.20)
	90-94	3,379,961	2,167	73		1.00 (Reference)	2,067,005	571	33		1.00 (Reference)
	95-99	2,647,841	1,658	72	0.311	0.97 (0.91-1.03)	1,364,378	409	38	0.245	1.08 (0.95-1.22)
	100-104	1,628,404	1,104	85	0.369	1.03 (0.96-1.11)	690,653	215	31	0.212	1.11 (0.94-1.29)
	105-109	1,019,857	703	91	0.441	1.03 (0.95-1.13)	361,706	117	97	0.193	1.14 (0.94-1.39)
	110-117	783,818	645	97	<0.001	1.21 (1.11-1.32)	239,403	82	79	0.130	1.20 (0.95-1.51)
	118-125	377,702	371	69	<0.001	1.41 (1.27-1.58)	96,713	40	37	0.028	1.43 (1.04-1.98)
	126-139	199,528	194	94	<0.001	1.38 (1.19-1.60)	45,186	18	37	0.188	1.37 (0.86-2.19)
	140-169	145,001	160	147	<0.001	1.54 (1.31-1.81)	31,253	13	30	0.226	1.40 (0.81-2.43)
	170-200	45,206	58	81	<0.001	1.77 (1.36-2.30)	9,405	6	76	0.074	2.08 (0.93-4.66)
	≥200	65,853	143	234	<0.001	3.05 (2.57-3.61)	15,750	21	106	<0.001	4.33 (2.79-6.70)
35-44	<65	169,590	311	152	0.001	1.22 (1.09-1.37)	104,849	102	105	0.018	1.27 (1.04-1.56)
	65-69	276,203	394	193	0.444	0.96 (0.87-1.06)	181,763	147	62	0.486	1.06 (0.90-1.26)
	70-74	1,569,494	2,436	187	0.049	1.05 (1.00-1.10)	1,066,458	840	96	0.405	1.04 (0.95-1.13)
	75-79	2,303,436	3,510	165	0.102	1.04 (0.99-1.08)	1,582,610	1,180	83	0.632	0.98 (0.91-1.06)
	80-84	2,768,741	4,135	183	0.323	1.02 (0.98-1.07)	2,000,243	1,494	87	0.429	0.97 (0.90-1.04)
	85-89	3,187,194	4,682	179	0.816	1.00 (0.96-1.05)	2,232,438	1,670	97	0.337	0.97 (0.90-1.04)
	90-94	3,099,955	4,536	163		1.00 (Reference)	1,955,557	1,537	89		1.00 (Reference)
	95-99	2,672,240	3,900	179	0.591	0.99 (0.95-1.03)	1,453,344	1,197	99	0.376	1.03 (0.96-1.12)
	100-104	1,882,498	3,117	180	<0.001	1.10 (1.05-1.15)	880,720	723	99	0.781	1.01 (0.93-1.11)
	105-109	1,292,728	2,253	202	<0.001	1.14 (1.08-1.19)	514,122	446	122	0.347	1.05 (0.95-1.17)
	110-117	1,092,431	2,206	252	<0.001	1.28 (1.22-1.35)	380,449	397	135	<0.001	1.24 (1.11-1.39)
	118-125	590,693	1,389	267	<0.001	1.45 (1.37-1.54)	176,829	204	156	<0.001	1.36 (1.17-1.57)
	126-139	349,425	911	339	<0.001	1.59 (1.48-1.70)	88,782	110	158	<0.001	1.43 (1.18-1.74)
	140-169	311,437	940	358	<0.001	1.80 (1.68-1.93)	73,018	115	207	<0.001	1.79 (1.48-2.16)
	170-200	124,300	435	341	<0.001	2.07 (1.88-2.29)	28,212	36	81	0.027	1.45 (1.04-2.02)
	≥200	186,795	980	576	<0.001	3.11 (2.90-3.33)	42,988	104	308	<0.001	2.78 (2.28-3.40)
45-54	<65	96,440	483	630	<0.001	1.24 (1.13-1.36)	90,617	154	211	0.026	1.20 (1.02-1.41)

Age group, years	FSG group, mg/dL	Men (n=7,090,089)					Women (n=5,365,272)				
		Person year	No. of death	ASR[b]	p-value	HR (95% CI)	Person year	No. of death	ASR[b]	p-value	HR (95% CI)
	65-69	149,078	699	627	<0.001	1.19 (1.10-1.29)	156,721	228	155	0.634	1.03 (0.90-1.18)
	70-74	885,719	3,513	520	0.190	1.03 (0.99-1.07)	918,594	1,408	168	0.003	1.10 (1.03-1.17)
	75-79	1,303,374	5,089	460	0.120	1.03 (0.99-1.07)	1,397,248	2,058	160	0.039	1.06 (1.00-1.12)
	80-84	1,635,948	6,306	484	0.109	1.03 (0.99-1.06)	1,843,369	2,656	214	0.199	1.04 (0.98-1.09)
	85-89	1,946,425	7,298	449	0.643	1.01 (0.98-1.04)	2,147,481	3,027	172	0.760	1.01 (0.96-1.06)
	90-94	1,986,767	7,316	529		1.00 (Reference)	2,025,160	2,848	181		1.00 (Reference)
	95-99	1,807,390	6,872	499	0.037	1.04 (1.00-1.07)	1,604,444	2,346	177	0.340	1.03 (0.97-1.08)
	100-104	1,384,637	5,463	491	<0.001	1.07 (1.04-1.11)	1,060,642	1,610	207	0.118	1.05 (0.99-1.12)
	105-109	1,016,017	4,370	572	<0.001	1.16 (1.11-1.20)	671,636	1,162	212	<0.001	1.18 (1.10-1.26)
	110-117	945,298	4,425	568	<0.001	1.24 (1.19-1.29)	546,410	1,024	203	<0.001	1.25 (1.16-1.34)
	118-125	551,679	2,880	636	<0.001	1.35 (1.29-1.41)	288,293	618	259	<0.001	1.41 (1.29-1.54)
	126-139	357,630	2,108	753	<0.001	1.50 (1.43-1.58)	166,916	342	190	<0.001	1.32 (1.18-1.48)
	140-169	345,020	2,359	858	<0.001	1.71 (1.63-1.79)	142,127	336	388	<0.001	1.51 (1.35-1.69)
	170-200	148,950	1,141	1,034	<0.001	1.89 (1.78-2.01)	54,534	158	363	<0.001	1.86 (1.58-2.18)
	≥200	218,154	2,370	1,356	<0.001	2.70 (2.58-2.83)	82,138	373	548	<0.001	2.91 (2.61-3.24)
55-64	<65	60,126	894	1,889	<0.001	1.25 (1.17-1.34)	58,581	294	585	0.008	1.17 (1.04-1.32)
	65-69	87,952	1,197	2,124	<0.001	1.18 (1.11-1.25)	95,019	430	770	0.196	1.07 (0.97-1.18)
	70-74	508,796	6,158	1,757	<0.001	1.08 (1.05-1.12)	529,392	2,218	763	0.262	1.03 (0.98-1.08)
	75-79	728,245	8,311	1,705	0.003	1.04 (1.01-1.07)	804,431	3,380	693	0.091	1.04 (0.99-1.08)
	80-84	932,736	10,131	1,611	0.264	1.02 (0.99-1.04)	1,094,876	4,532	573	0.080	1.04 (1.00-1.08)
	85-89	1,112,916	11,625	1,532	0.348	0.99 (0.96-1.01)	1,328,291	5,321	609	0.812	1.00 (0.97-1.04)
	90-94	1,139,320	11,838	1,523		1.00 (Reference)	1,314,640	5,213	632		1.00 (Reference)
	95-99	1,059,488	11,058	1,551	0.163	1.02 (0.99-1.05)	1,122,375	4,635	646	0.065	1.04 (1.00-1.08)
	100-104	839,922	8,964	1,665	<0.001	1.05 (1.02-1.08)	813,453	3,454	638	0.011	1.06 (1.01-1.10)
	105-109	639,512	7,087	1,490	<0.001	1.09 (1.06-1.12)	575,612	2,659	715	<0.001	1.14 (1.09-1.20)
	110-117	626,003	7,486	1,878	<0.001	1.17 (1.13-1.20)	519,922	2,500	702	<0.001	1.17 (1.12-1.23)
	118-125	398,717	5,285	1,833	<0.001	1.28 (1.24-1.33)	306,926	1,635	726	<0.001	1.28 (1.21-1.35)
	126-139	269,932	3,934	1,999	<0.001	1.41 (1.36-1.46)	194,409	1,117	1,015	<0.001	1.38 (1.29-1.47)
	140-169	262,647	4,395	2,454	<0.001	1.61 (1.55-1.66)	175,620	1,134	939	<0.001	1.54 (1.44-1.64)
	170-200	112,257	2,089	2,608	<0.001	1.78 (1.70-1.87)	65,686	531	1,070	<0.001	1.92 (1.75-2.10)
	≥200	154,574	3,826	3,296	<0.001	2.38 (2.30-2.47)	94,390	1,081	1,664	<0.001	2.70 (2.53-2.88)
65-74	<65	32,275	1,371	5,773	<0.001	1.23 (1.17-1.30)	33,915	634	2,788	<0.001	1.22 (1.12-1.32)
	65-69	43,811	1,594	5,319	0.003	1.08 (1.03-1.14)	51,411	868	3,212	<0.001	1.13 (1.06-1.21)
	70-74	233,268	8,217	5,975	<0.001	1.08 (1.05-1.11)	283,168	4,387	3,296	0.001	1.06 (1.02-1.10)
	75-79	320,489	10,643	6,053	0.002	1.04 (1.02-1.07)	404,590	6,130	2,752	0.003	1.05 (1.02-1.09)

Age group, years	FSG group, mg/dL	Men (n=7,090,089)					Women (n=5,365,272)				
		Person year	No. of death	ASR[b]	p-value	HR (95% CI)	Person year	No. of death	ASR[b]	p-value	HR (95% CI)
	80-84	410,628	13,109	5,188	0.052	1.02 (1.00-1.05)	538,223	7,868	2,751	0.048	1.03 (1.00-1.06)
	85-89	484,597	15,149	5,006	0.191	1.02 (0.99-1.04)	650,456	9,172	2,522	0.908	1.00 (0.97-1.03)
	90-94	489,806	14,769	4,623		1.00 (Reference)	658,325	9,215	2,528		1.00 (Reference)
	95-99	444,095	13,535	5,063	0.056	1.02 (1.00-1.05)	578,920	8,358	2,685	0.065	1.03 (1.00-1.06)
	100-104	363,336	11,224	5,776	<0.001	1.05 (1.02-1.07)	444,396	6,704	2,657	<0.001	1.07 (1.04-1.11)
	105-109	275,076	8,877	5,281	<0.001	1.10 (1.07-1.13)	329,784	5,200	2,340	<0.001	1.11 (1.07-1.15)
	110-117	274,607	9,322	5,365	<0.001	1.16 (1.13-1.19)	323,508	5,564	2,914	<0.001	1.21 (1.17-1.25)
	118-125	180,539	6,517	6,620	<0.001	1.23 (1.19-1.27)	211,542	3,853	2,961	<0.001	1.27 (1.22-1.32)
	126-139	124,836	4,703	5,525	<0.001	1.31 (1.26-1.35)	139,122	2,684	3,196	<0.001	1.35 (1.30-1.41)
	140-169	121,154	5,162	6,545	<0.001	1.45 (1.41-1.50)	131,553	2,828	3,733	<0.001	1.50 (1.44-1.56)
	170-200	47,681	2,281	5,378	<0.001	1.66 (1.58-1.73)	48,973	1,310	4,146	<0.001	1.88 (1.77-1.99)
	≥200	60,670	3,505	9,283	<0.001	2.05 (1.97-2.12)	65,743	2,141	4,975	<0.001	2.32 (2.21-2.43)
75-99	<65	5,941	639	10,912	<0.001	1.19 (1.10-1.29)	7,739	463	5,708	0.307	1.05 (0.96-1.15)
	65-69	9,263	861	9,026	0.375	1.03 (0.96-1.11)	11,275	696	5,906	0.006	1.11 (1.03-1.20)
	70-74	45,279	4,292	9,418	0.001	1.07 (1.03-1.11)	61,044	3,497	5,471	0.040	1.04 (1.00-1.09)
	75-79	61,539	5,636	9,088	0.004	1.05 (1.02-1.09)	86,597	4,768	5,265	0.557	1.01 (0.97-1.05)
	80-84	77,124	6,656	8,476	0.355	0.98 (0.95-1.02)	112,432	6,086	5,163	0.805	1.00 (0.96-1.03)
	85-89	92,936	7,830	8,278	0.098	0.97 (0.94-1.00)	134,749	7,231	5,087	0.882	1.00 (0.97-1.04)
	90-94	92,919	7,880	8,335		1.00 (Reference)	135,939	7,314	5,090		1.00 (Reference)
	95-99	83,813	6,989	8,195	0.356	0.98 (0.95-1.02)	123,993	6,718	5,130	0.356	1.02 (0.98-1.05)
	100-104	66,369	5,811	8,609	0.011	1.04 (1.01-1.08)	100,311	5,465	5,154	0.299	1.02 (0.98-1.06)
	105-109	52,411	4,690	8,823	<0.001	1.08 (1.04-1.12)	79,286	4,507	5,386	0.003	1.06 (1.02-1.10)
	110-117	52,459	4,874	9,201	<0.001	1.12 (1.08-1.16)	82,354	4,982	5,729	<0.001	1.14 (1.10-1.18)
	118-125	37,039	3,620	9,754	<0.001	1.17 (1.12-1.22)	59,653	3,681	5,830	<0.001	1.16 (1.11-1.20)
	126-139	24,674	2,542	10,184	<0.001	1.26 (1.20-1.31)	40,823	2,647	6,115	<0.001	1.22 (1.16-1.27)
	140-169	24,532	2,725	11,063	<0.001	1.33 (1.27-1.39)	39,208	2,771	6,758	<0.001	1.35 (1.30-1.42)
	170-200	9,333	1,084	11,687	<0.001	1.45 (1.36-1.54)	13,850	1,107	7,713	<0.001	1.56 (1.46-1.66)
	≥200	10,987	1,383	12,852	<0.001	1.64 (1.54-1.73)	18,083	1,636	8,976	<0.001	1.85 (1.75-1.95)

ASR, age standardized death rate per 100,000 person-years; CI, confidence interval; FSG, fasting serum glucose; HR, hazard ratio

[a] HRs were calculated by Cox models stratified by age (baseline age, years: 18-24, 25-34, 35-44, 45-54, 55-64, 65-74, 75-84, 85-99), after adjustment for age at baseline, sex (if applicable), smoking status, alcohol use, physical activity, body mass index, systolic blood pressure, and total cholesterol.

[b] Uniformly age-standardized death rate per 100,000 person-years (namely, the simple mean of calculable age-specific rates in the 14 age-specific rates at ages 18-24 years, and 25-29 years to 85 years or older by 5 years based on attained age during follow-up) was calculated for each FSG category. Several age-specific rates were calculated at each age group, and age-specific rate in the oldest ages at each age group can be very high due to small number of follow-up years. Therefore, death rate at a FSG category can be very high compared to other categories.

To convert glucose from mg/dL to mmol/L, multiply by 0.0555.

Table S6. HRs[a] for death associated with four categories of fasting glucose

Age group, years	FSG group, mg/dL	ADA classification: Men and women p-value	HR (95% CI)		Men p-value	HR (95% CI)		Women p-value	HR (95% CI)		WHO classification: FSG group, mg/dL	Men and women p-value	HR (95% CI)	
18-99	<70	<0.001	1.12	(1.11-1.14)	<0.001	1.13	(1.11-1.16)	<0.001	1.10	(1.07-1.14)	<70	<0.001	1.11	(1.09-1.13)
all ages	70-99		1.00	(Reference)		1.00	(Reference)		1.00	(Reference)	70-109		1.00	(Reference)
combined	100-125	<0.001	1.12	(1.11-1.13)	<0.001	1.12	(1.11-1.12)	<0.001	1.11	(1.10-1.12)	110-125	<0.001	1.19	(1.18-1.20)
	≥126	<0.001	1.60	(1.59-1.61)	<0.001	1.61	(1.59-1.62)	<0.001	1.54	(1.52-1.57)	≥126	<0.001	1.58	(1.57-1.59)
18-34	<70	0.102	1.07	(0.99-1.15)	0.120	1.07	(0.98-1.17)	0.675	1.03	(0.89-1.20)	<70	0.145	1.06	(0.98-1.14)
	70-99		1.00	(Reference)		1.00	(Reference)		1.00	(Reference)	70-109		1.00	(Reference)
	100-125	<0.001	1.14	(1.10-1.18)	<0.001	1.15	(1.10-1.20)	0.103	1.08	(0.98-1.20)	110-125	<0.001	1.30	(1.22-1.38)
	≥126	<0.001	1.77	(1.63-1.92)	<0.001	1.77	(1.63-1.93)	<0.001	1.81	(1.39-2.35)	≥126	<0.001	1.76	(1.62-1.91)
35-44	<70	0.029	1.07	(1.01-1.15)	0.235	1.05	(0.97-1.13)	0.032	1.15	(1.01-1.30)	<70	0.079	1.06	(0.99-1.13)
	70-99		1.00	(Reference)		1.00	(Reference)		1.00	(Reference)	70-109		1.00	(Reference)
	100-125	<0.001	1.17	(1.14-1.20)	<0.001	1.18	(1.15-1.21)	<0.001	1.11	(1.05-1.17)	110-125	<0.001	1.30	(1.26-1.34)
	≥126	<0.001	1.96	(1.90-2.03)	<0.001	1.98	(1.91-2.06)	<0.001	1.80	(1.62-2.00)	≥126	<0.001	1.93	(1.87-2.00)
45-54	<70	<0.001	1.16	(1.10-1.22)	<0.001	1.19	(1.12-1.26)	0.238	1.06	(0.96-1.18)	<70	<0.001	1.14	(1.08-1.20)
	70-99		1.00	(Reference)		1.00	(Reference)		1.00	(Reference)	70-109		1.00	(Reference)
	100-125	<0.001	1.15	(1.13-1.17)	<0.001	1.15	(1.13-1.17)	<0.001	1.13	(1.10-1.17)	110-125	<0.001	1.24	(1.21-1.27)
	≥126	<0.001	1.82	(1.78-1.86)	<0.001	1.84	(1.79-1.88)	<0.001	1.69	(1.59-1.79)	≥126	<0.001	1.79	(1.75-1.83)
55-64	<70	<0.001	1.16	(1.12-1.20)	<0.001	1.19	(1.14-1.24)	0.033	1.08	(1.01-1.17)	<70	<0.001	1.15	(1.11-1.19)
	70-99		1.00	(Reference)		1.00	(Reference)		1.00	(Reference)	70-109		1.00	(Reference)
	100-125	<0.001	1.11	(1.10-1.12)	<0.001	1.11	(1.09-1.12)	<0.001	1.11	(1.09-1.14)	110-125	<0.001	1.18	(1.16-1.20)
	≥126	<0.001	1.69	(1.66-1.72)	<0.001	1.68	(1.65-1.71)	<0.001	1.70	(1.64-1.76)	≥126	<0.001	1.67	(1.64-1.70)
65-74	<70	<0.001	1.13	(1.10-1.16)	<0.001	1.12	(1.08-1.16)	<0.001	1.14	(1.08-1.20)	<70	<0.001	1.12	(1.09-1.15)
	70-99		1.00	(Reference)		1.00	(Reference)		1.00	(Reference)	70-109		1.00	(Reference)
	100-125	<0.001	1.10	(1.09-1.12)	<0.001	1.09	(1.08-1.11)	<0.001	1.12	(1.10-1.14)	110-125	<0.001	1.17	(1.15-1.18)
	≥126	<0.001	1.53	(1.51-1.55)	<0.001	1.49	(1.46-1.52)	<0.001	1.59	(1.56-1.63)	≥126	<0.001	1.52	(1.49-1.54)
75-99	<70	<0.001	1.09	(1.05-1.13)	0.001	1.09	(1.03-1.15)	0.011	1.08	(1.02-1.14)	<70	<0.001	1.08	(1.04-1.12)
	70-99		1.00	(Reference)		1.00	(Reference)		1.00	(Reference)	70-109		1.00	(Reference)
	100-125	<0.001	1.08	(1.07-1.10)	<0.001	1.09	(1.07-1.11)	<0.001	1.08	(1.06-1.09)	110-125	<0.001	1.13	(1.11-1.15)
	≥126	<0.001	1.38	(1.36-1.40)	<0.001	1.36	(1.33-1.39)	<0.001	1.39	(1.36-1.43)	≥126	<0.001	1.37	(1.34-1.39)

ADA, American Diabetes Association; CI, confidence interval; FSG, fasting serum glucose; HR, hazard ratio

[a] HRs were calculated by Cox models stratified by age (baseline age, years: 18-24, 25-34, 35-44, 45-54, 55-64, 65-74, 75-84, 85-99), after adjustement for age at baseline, sex (if applicable), smoking status, alcohol use, physical activity, body mass index, systolic blood pressure, and total cholesterol.

To convert glucose from mg/dL to mmol/L, multiply by 0.0555.
